# Fault Detection of Electric Impact Drills and Coffee Grinders Using Acoustic Signals

**DOI:** 10.3390/s19020269

**Published:** 2019-01-11

**Authors:** Adam Glowacz

**Affiliations:** Department of Automatic Control and Robotics, Faculty of Electrical Engineering, Automatics, Computer Science and Biomedical Engineering, AGH University of Science and Technology, al. A. Mickiewicza 30, 30-059 Kraków, Poland; adglow@agh.edu.pl

**Keywords:** motor, mechanical fault, detection, RMS, sound, drill, safety, pattern, bearing, fan, shaft

## Abstract

Increasing demand for higher safety of motors can be noticed in recent years. Developing of new fault detection techniques is related with higher safety of motors. This paper presents fault detection technique of an electric impact drill (EID), coffee grinder A (CG-A), and coffee grinder B (CG-B) using acoustic signals. The EID, CG-A, and CG-B use commutator motors. Measurement of acoustic signals of the EID, CG-A, and CG-B was carried out using a microphone. Five signals of the EID are analysed: healthy, with 15 broken rotor blades (faulty fan), with a bent spring, with a shifted brush (motor off), with a rear ball bearing fault. Four signals of the CG-A are analysed: healthy, with a heavily damaged rear sliding bearing, with a damaged shaft and heavily damaged rear sliding bearing, motor off. Three acoustic signals of the CG-B are analysed: healthy, with a light damaged rear sliding bearing, motor off. Methods such as: Root Mean Square (RMS), MSAF-17-MULTIEXPANDED-FILTER-14 are used for feature extraction. The MSAF-17-MULTIEXPANDED-FILTER-14 method is also developed and described in the paper. Classification is carried out using the Nearest Neighbour (NN) classifier. An acoustic based analysis is carried out. The results of the developed method MSAF-17-MULTIEXPANDED-FILTER-14 are very good (total efficiency of recognition of all classes—TE_D_ = 96%, TE_CG-A_ = 97%, TE_CG-B_ = 100%).

## 1. Introduction

Today rotating machinery is used for a wide variety of industrial applications such as electrical motors, engines, home appliances and electric power tools. It can also find applications in mining, oil, car, energy, and the steel industry. Cost-effective and non-destructive fault detection is profitable for industry. It can be used for rotating machinery. Reliable operation of rotating machinery is essential for many factories, oil refineries, industrial plants. Gas turbines, motors, pumps, aircraft engines, drive trains can be diagnosed by fault diagnosis techniques. Machines must operate safely without interruptions. If faults occur, the consequences can be catastrophic. Damaged machines generate costs, for example replacement of the machine or stopped production lines in the factory. Thus, the benefits of fault detection are maintenance cost savings.

There are lots of studies in the literature related to fault diagnosis and fault detection of rotating machinery. Analysis of electric currents is developed in the articles [1,2,3,4,5]. The results of current recognition are very good. However it can only be used for limited number of electrical faults such as broken bars, shorted rotors, stator coils. Electric current-based methods are usually useless for many mechanical faults such as damaged teeth on sprockets, faulty gears, faulty fans, etc. The next methods developed in the literature are based on vibration analysis [6,7,8,9,10,11,12,13] and acoustic analysis [14,15,16,17,18,19,20,21,22]. They are very effective. There is no need to connect a measuring sensor with the machine for acoustic-based measurements. Vibration-based measurements require a connection between the sensor and the machine. Vibration signals are less noisy than acoustic signals. Both of them can measure signals immediately. Vibration and acoustic analysis can also detect mechanical and electrical faults of rotating machinery.

The next method of fault detection is thermal analysis. Thermal analysis methods are described in [23,24,25]. Temperature detection can be performed using thermal imaging cameras, infrared thermometers and portable laser thermometers. If we use a thermal imaging camera or portable laser thermometer, then we can measure from a distance. The next method of fault detection of rotating machinery is oil analysis. It can provide diagnostic information about the condition of rotating machinery. In [26,27] some methods are mentioned: rotating disc electrode spectroscopy, inductively coupled plasma spectroscopy, FPQ-XRF, acid digestion, light blocking, light scattering, laser imaging, laser imaging, ferrography, light blocking, light scattering, laser imaging, fuel sniffer, gas chromatography, gravimetric, Karl Fischer titration, viscosity, etc. Multidimensional prognostics for rotating machinery was also presented [28]. 

**Figure 1:**
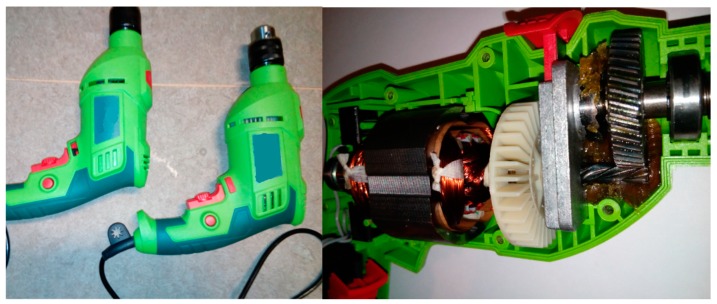
Healthy EID.

**Figure 2:**
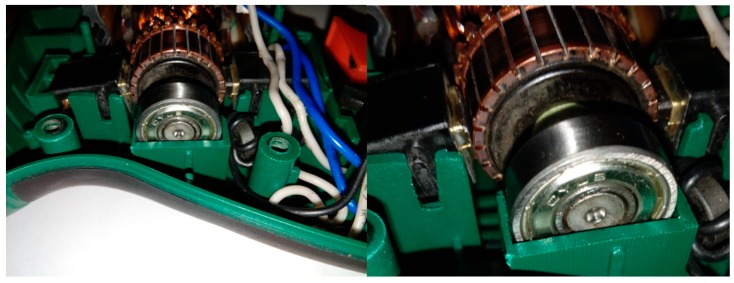
Healthy EID (EID with a healthy rear bearing).

**Figure 3:**
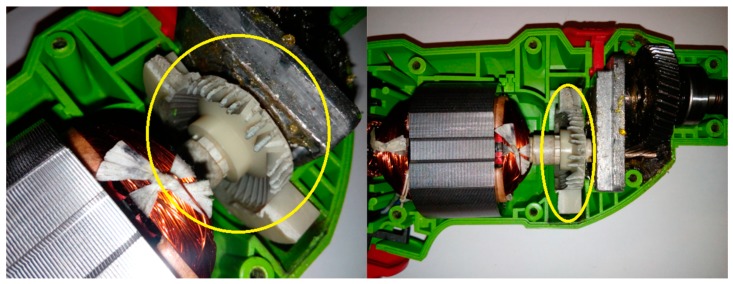
EID with 15 broken rotor blades (indicated by yellow circle).

**Figure 4:**
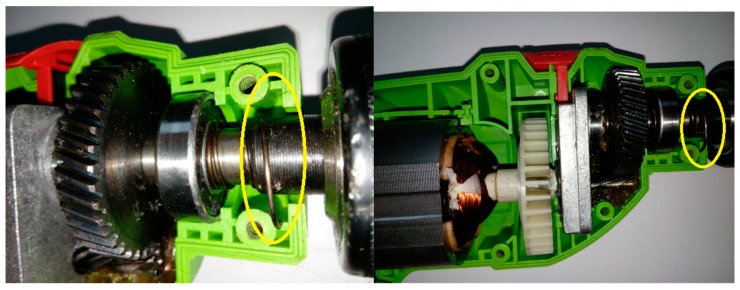
EID with a bent spring (indicated by yellow circle).

**Figure 5:**
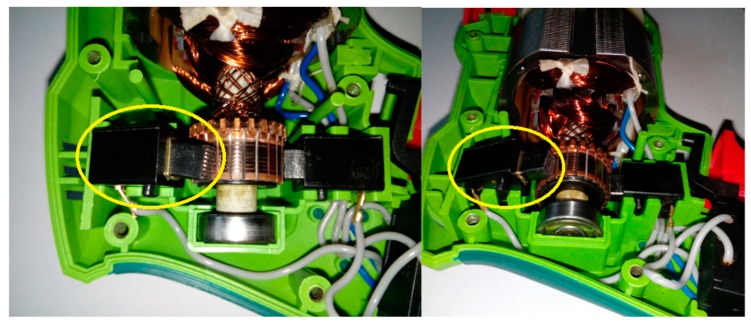
EID with a shifted brush (indicated by yellow circle).

**Figure 6:**
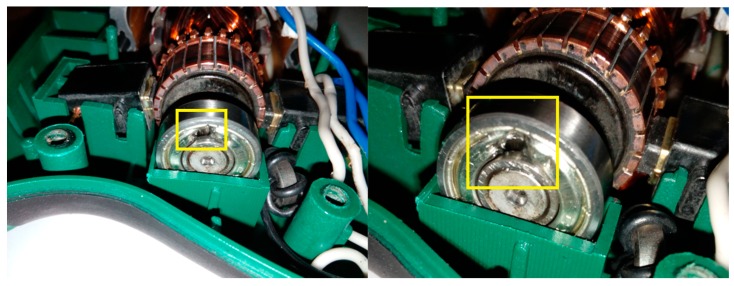
EID with a rear ball bearing fault (indicated by yellow square).

This article describes the application of the acoustic-based approach to an electric impact drill (EID)—Verto 50G515, made in China, and two coffee grinders designated as coffee grinder A (CG-A)—Metrox ME-1497, made in China, and coffee grinder B (CG-B)—Sencor SCG 1050WH, made in China. The EID, CG-A, and CG-B use commutator motors. The commutator motor is a type of electrical motor used for power tools and home appliances such as blenders, coffee grinders and hair driers. The author analysed five electric impact drills (one healthy and four faulty). Each of them generates acoustic signals. Five signals are analysed: healthy (Figure 1 and Figure 2), with 15 broken rotor blades (faulty fan) (Figure 3), with a bent spring (Figure 4), with a shifted brush (Figure 5), with a rear ball bearing fault (Figure 6).

**Figure 7:**
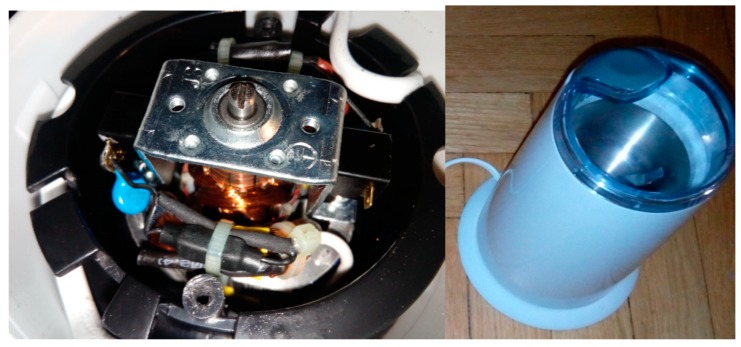
Healthy CG-A.

**Figure 8:**
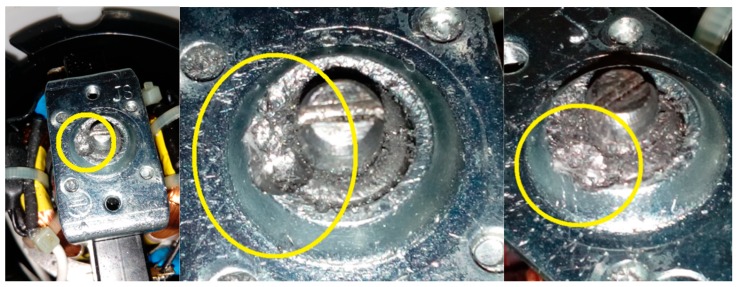
CG-A with a heavily damaged rear sliding bearing (indicated by yellow circle).

**Figure 9:**
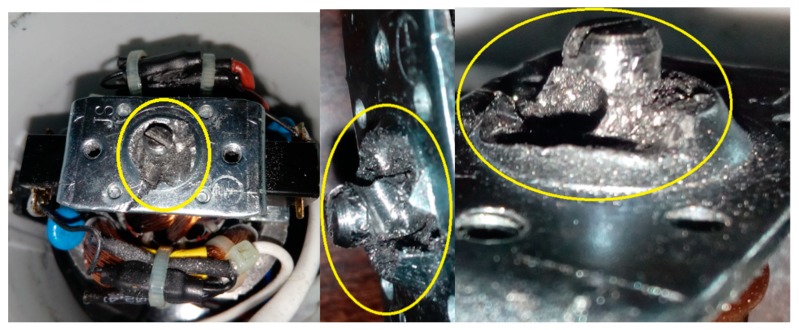
CG-A with a damaged shaft and heavily damaged rear sliding bearing (indicated by yellow circle).

**Figure 10:**
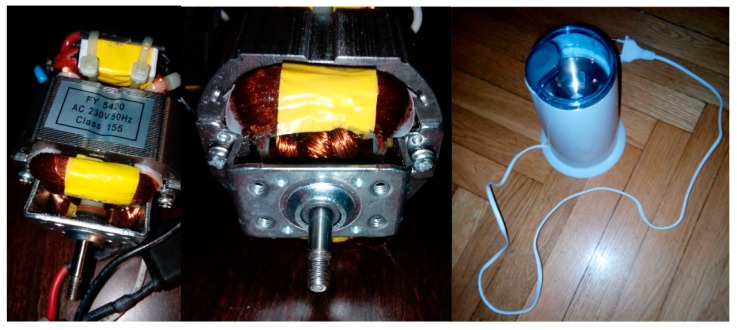
Motor off (CG-A off).

Four signals of the CG-A were analysed: healthy CG-A (Figure 7), CG-A with a heavily damaged rear sliding bearing (Figure 8), CG-A with a damaged shaft and heavily damaged rear sliding bearing (Figure 9), motor off (Figure 10).

**Figure 11:**
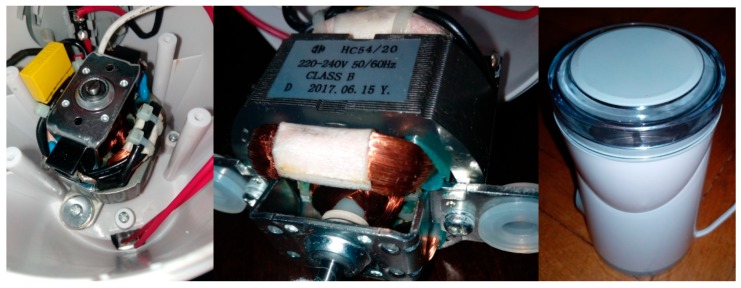
Healthy CG-B.

**Figure 12:**
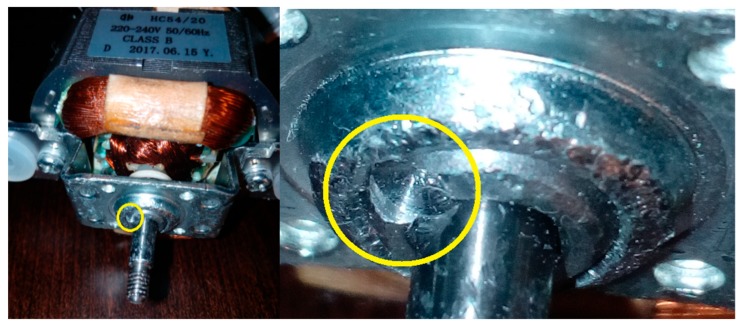
CG-B with a light damaged rear sliding bearing (indicated by yellow circle).

**Figure 13:**
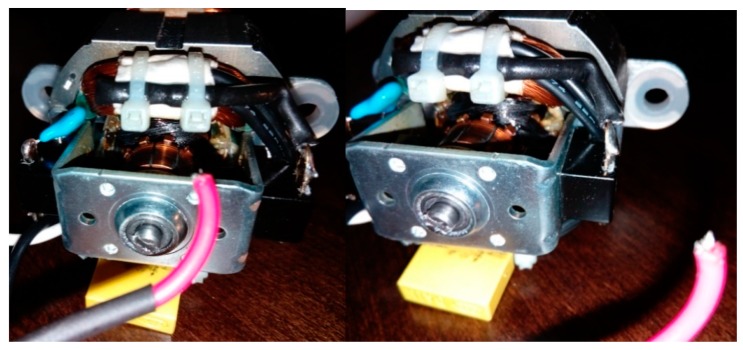
Motor off (CG-B off).

Three signals of the CG-B were analysed: healthy CG-B (Figure 11), CG-B with a light damaged rear sliding bearing (Figure 12), motor off (Figure 13).

In Section 1, the author presents a review of the fault detection methods. In Section 2, the author describes the acoustic based approach and proposed methods of signal processing. In Section 3, the recognition results of the EID, CG-A, and CG-B are presented. A discussion is presented in Section 4. In Section 5, summary and conclusions are described.

## 2. Developed Acoustic Based Approach

**Figure 14:**
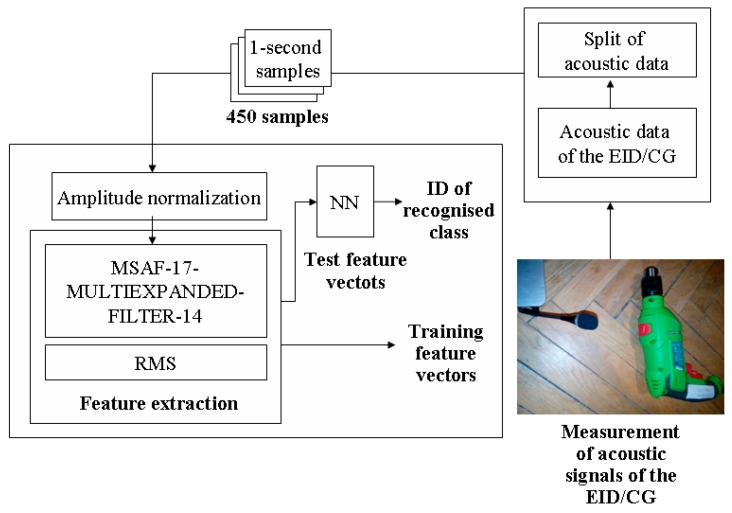
Developed acoustic based approach.

The developed acoustic-based approach used signal processing methods and the acoustic data of the EID, CG-A and CG-B. Acoustic data were obtained using a HAMA 00057152 microphone. The parameters of the microphone are: frequency response 30–16,000 Hz, rated impedance 1400 Ω, sensitivity −62 dB. The microphone was placed 0.2–0.3 m away from the EID, CG-A and CG-B. Other types of microphones could be also used. Acoustic data were split (using “MPlayer library—The Movie Player”—wav file parameters sampling frequency 44,100 Hz, single channel, 16 bits resolution, stationary signal) and normalized. Normalization of amplitude divided each sample (in the time domain) by the maximum value of the signal (in time domain). After that feature vectors were formed using the RMS or MSAF-17-MULTIEXPANDED-FILTER-14 (the methodology is presented in Section 2.1). Next the Nearest Neighbour (NN) classifier compared feature vectors in the classification step. The developed acoustic based approach is shown in Figure 14.

**Figure 15:**
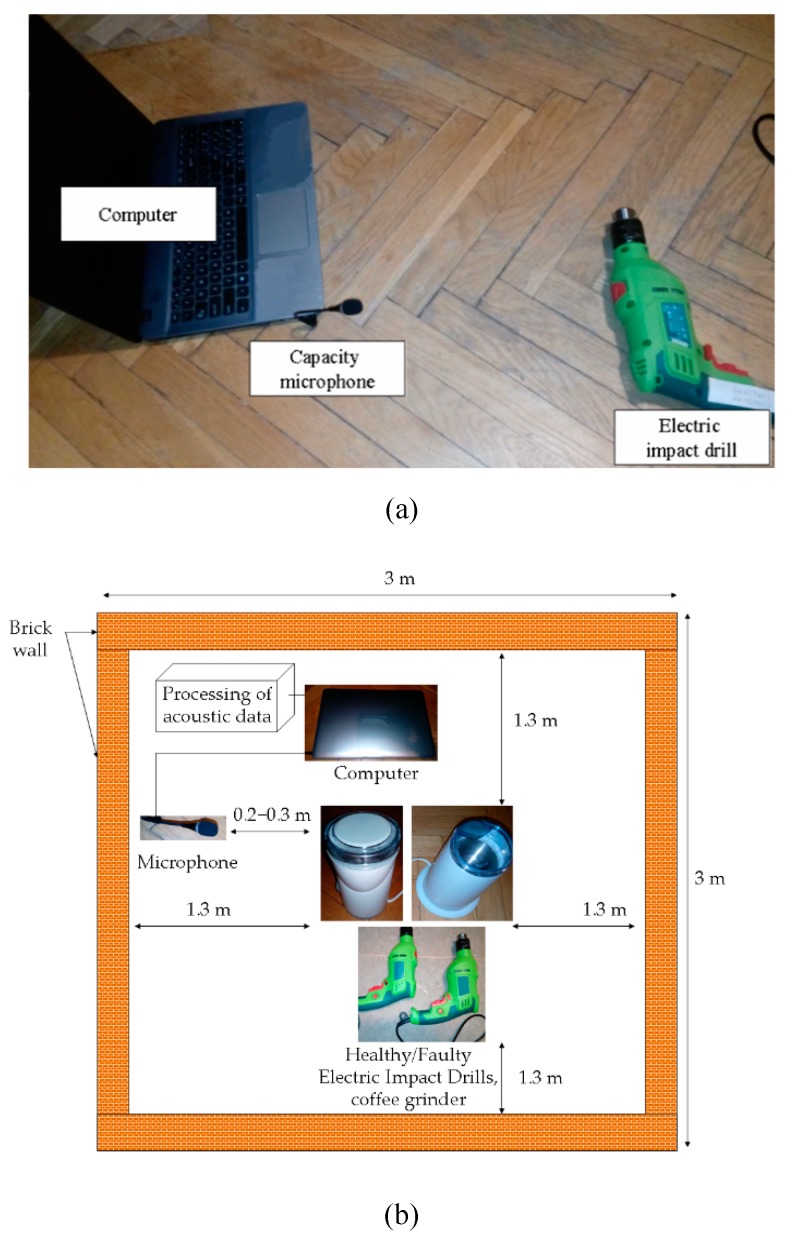
(**a**) Capacity microphone, computer and electric impact drill. (**b**) Measurement of acoustic signals.

An experimental setup consisted of the microphone and a computer. It was used to analyse the electric impact drill/coffee grinder (Figure 15a). Measurement of acoustic signals is depicted in Figure 15b.

### 2.1. MSAF-17-MULTIEXPANDED-FILTER-14

The Method of Selection of Amplitudes of Frequency Multiexpanded Filter (MSAF-17-MULTIEXPANDED-FILTER-14) was developed and implemented. This feature extraction method used differences between FFT spectra. It consists of seven signal processing steps:

(1) Compute Fast Fourier Transform (FFT) spectra for all states of the EID (for all training vectors). In the presented acoustic based approach the FFT provided a vector of 16384-elements. For 16,384 frequency components, the frequency spectrum is 22,050 Hz. Therefore, each frequency component is every 1.345 Hz. The computed vectors were defined as follows: healthy EID—**h** = [*h*_1_, *h*_2_, ..., *h*_16,384_], EID with 15 broken rotor blades (faulty fan)—**f** = [*f*_1_, *f*_2_, ..., *f*_16,384_], EID with a bent spring—**s** = [*s*_1_, *s*_2_, ..., *s*_16,384_], EID with a rear ball bearing fault—**b** = [*b*_1_, *b*_2_, ..., *b*_16,384_].
(2) For each training vector compute: **h** − **f**, **h** − **s**, **f** − **s**, **b** − **h**, **b** − **f**, **b** − **s**.
(3) Compute: |**h** − **f**|, |**h** − **s**|, |**f** − **s**|, |**b** − **h**|, |**b** − **f**|, |**b** − **s**|.
(4) Find 1–17 *Common Frequency Components* (*CFCs*) or set a parameter *Threshold of CFCs* (*ToCFCs)*. If there are no *CFCs*, then set a parameter *ToCFCs.* The parameter is defined as Equation (1):
  (1) $$ToCFCs=\frac{\begin{array}{cccc} Number & of & required & CFCs \end{array}}{\begin{array}{cccc} Number & of & all & differences \end{array}}$$

Let’s analyse the following example: three training sets are given. Each of them has four training samples. Eighteen differences are computed (six for the first training set, six for the second training set, six for the third training set). Let’s suppose that frequency component 130 Hz is found three times for |**h** − **f|.** Let’s suppose that frequency components 110, 160 Hz are found two times for |**h** − **s|**. Let’s suppose that frequency components 110, 140 Hz are found two times for |**f** − **s**|. Let’s suppose that frequency component 500 Hz is found three times for |**b** − **h**|. Let’s suppose that frequency components 600, 610 Hz are found two times for |**b** − **f**|. Let’s suppose that frequency components 600, 710 Hz are found two times for |**b** − **s**|. There are no *CFCs*. Only frequency components 110 Hz and 600 Hz are found four times. The MSAF-17-MULTIEXPANDED finds frequency components 110, 130, 140, 160, 500, 600, 610, 710 Hz, if *ToCFCs* is equal to 0.1111 (2/18). The MSAF-17-MULTIEXPANDED method finds 0 frequency components, if *ToCFCs* is equal to 0.2777 (5/18).

(5) Form groups of frequency components for a proper recognition. Considering the presented example, it can be noticed that the frequency component 110 Hz is good for |**h** − **s**| and |**f** − **s**|. The frequency component 130 Hz is good for |**h** − **f**|. The frequency component 500 Hz is good for |**b** − **h**|. The frequency component 600 Hz is good for |**b** − **f**| and |**b** − **s**|. The MSAF-17- MULTIEXPANDED-FILTER-14 finds 1 group consisted of 110, 130, 500, 600 Hz.
(6) Form bandwidths of frequency. Considering the presented example, 14 Hz bandwidths are selected. The MSAF-17-MULTIEXPANDED-FILTER-14 uses a value of 14 Hz. The value of 14 Hz is set experimentally. The middle of the first bandwidth is located at 110 Hz. The middle of the second bandwidth is located at 130 Hz. The middle of the third bandwidth is located at 500 Hz. The middle of the fourth bandwidth is located at 600 Hz. Following bandwidths are selected <103–117 Hz>, <123–137 Hz >, <493–507 Hz>, <593–607 Hz>.
(7) Using computed bandwidths, form a feature vector.

In other words, we can say that: 17—means that, we analyse 17 (local) maximum values of analysed difference between FFT spectra of acoustic signals, for example |**h** − **f**|, 14—means that, we set 14 Hz frequency bandwidth, for example for frequency 50 Hz it will be <50 − 7 Hz, 50 + 7Hz>.

**Figure 16:**
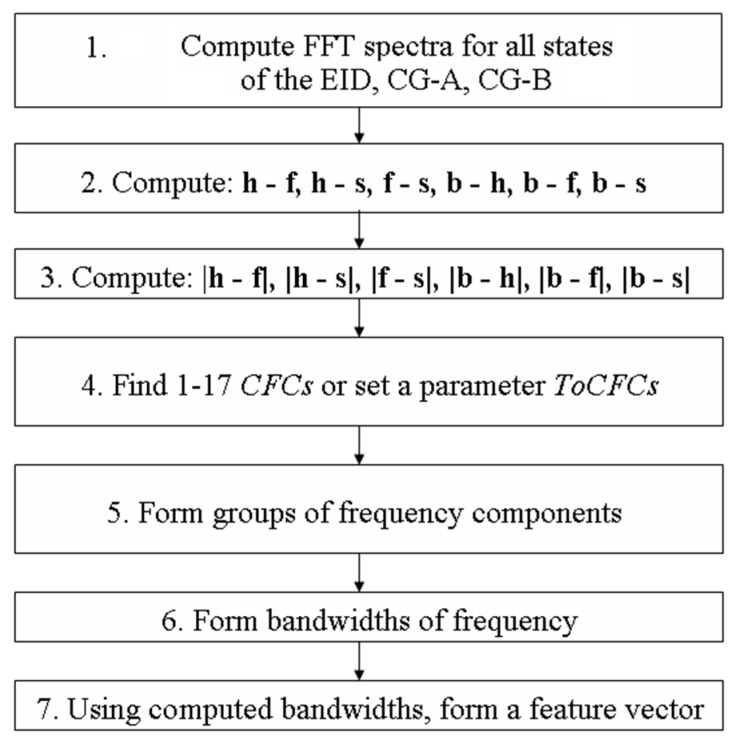
Block diagram of the developed method MSAF-17-MULTIEXPANDED-FILTER-14.

A block diagram of the developed method MSAF-17-MULTIEXPANDED-FILTER-14 is presented in Figure 16.

**Figure 17:**
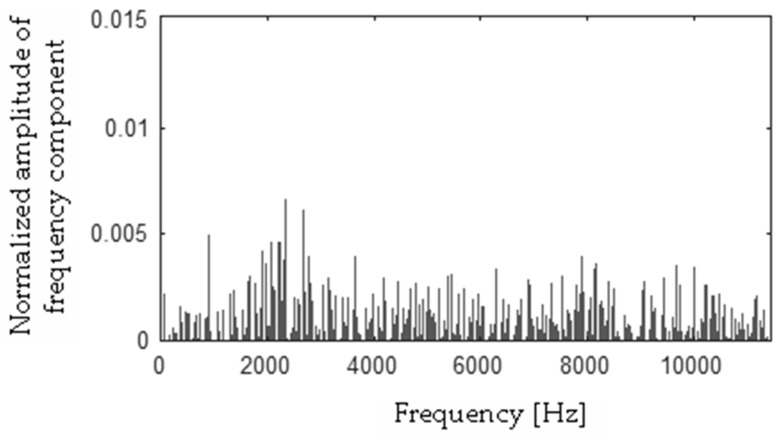
Difference (|**h** − **f**|) using the MSAF-17-MULTIEXPANDED-FILTER-14 method.

**Figure 18:**
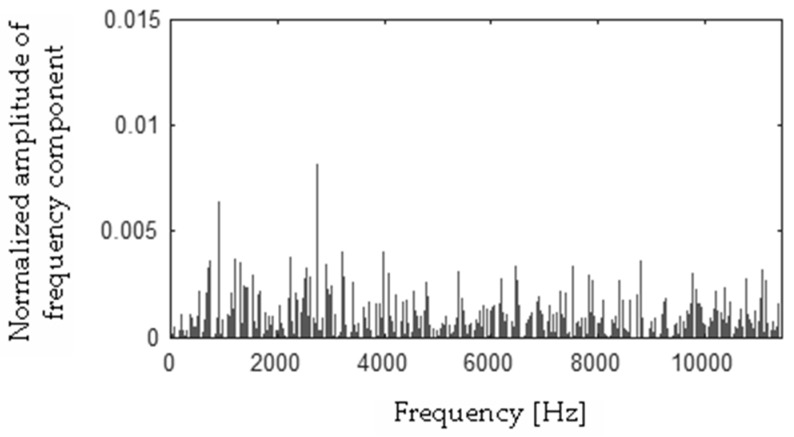
Difference (|**h** − **s**|) using the MSAF-17-MULTIEXPANDED-FILTER-14 method.

**Figure 19:**
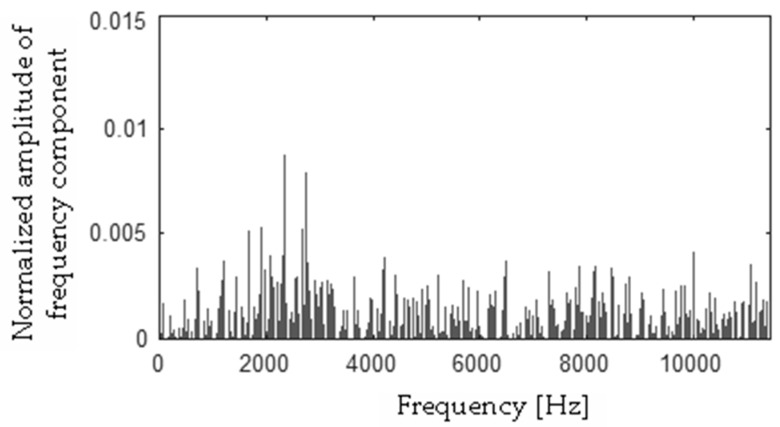
Difference (|**f** − **s**|) using the MSAF-17-MULTIEXPANDED-FILTER-14 method.

**Figure 20:**
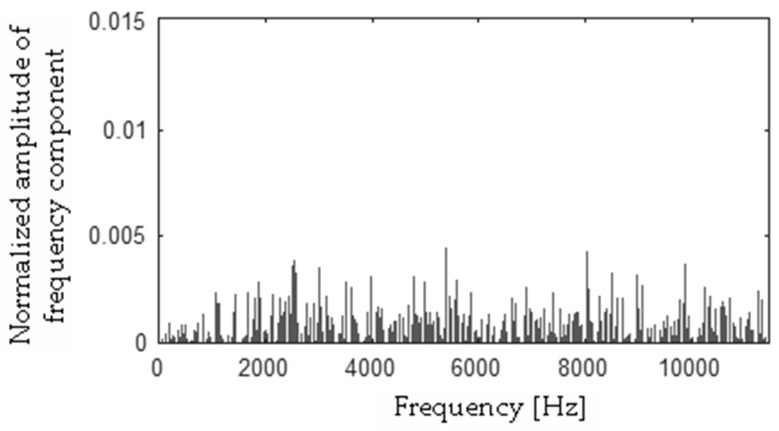
Difference (|**b** − **h**|) using the MSAF-17-MULTIEXPANDED-FILTER-14 method.

**Figure 21:**
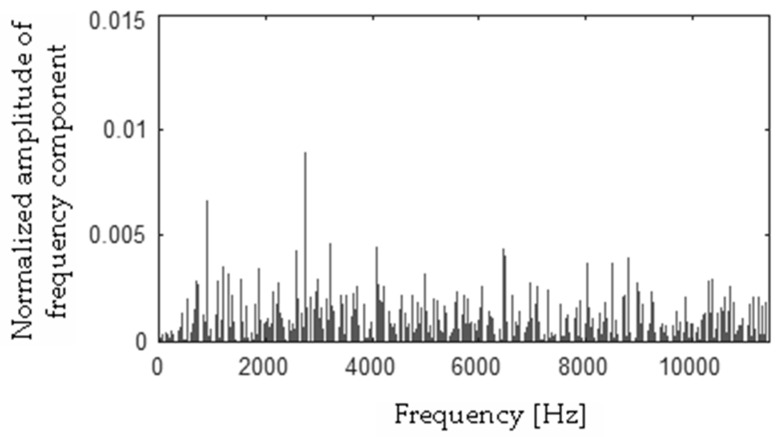
Difference (|**b** − **s**|) using the MSAF-17-MULTIEXPANDED-FILTER-14 method.

**Figure 22:**
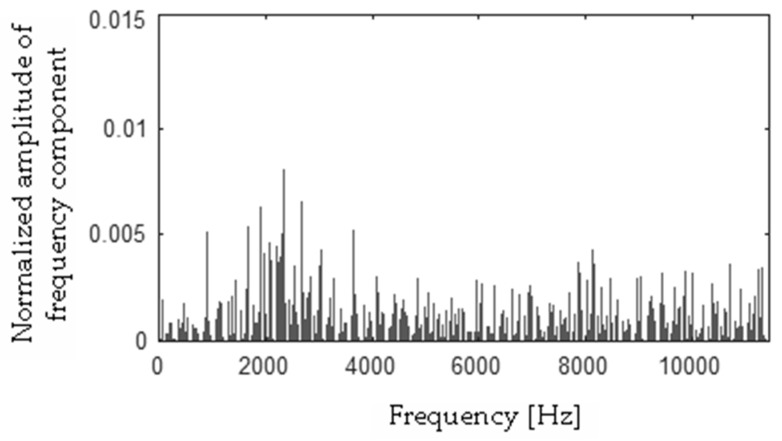
Difference (|**b** − **f**|) using the MSAF-17-MULTIEXPANDED-FILTER-14 method.

Differences between FFT spectra |**h** − **f**|, |**h** − **s**|, |**f** − **s**|, |**b** − **h**|, |**b** − **f**|, |**b** − **s**| were computed and are presented in Figure 17, Figure 18, Figure 19, Figure 20, Figure 21 and Figure 22.

The developed method MSAF-17-MULTIEXPANDED-FILTER-14 found the following frequency components: 278, 280, 457, 464, 468, 477, 479, 480, 481, 483, 557, 558, 2297, 2313, 2316, 2317, 11098, 11099, 11103, 11106, 11110, 11111, 11190, 11192, 11193, 11197, 11198, 11205, 11207, 11208, 11209, 11213, 11239, 11240, 11242, 11244, 11246 Hz.

Next the MSAF-17-MULTIEXPANDED-FILTER-14 selected seven frequency bandwidths of the EID: <271–287 Hz>, <450–490 Hz>, <550–565 Hz>, <2290–2324 Hz>, <11091–11118 Hz>, <11183–11220 Hz>, <11232–11253 Hz>.

**Figure 23:**
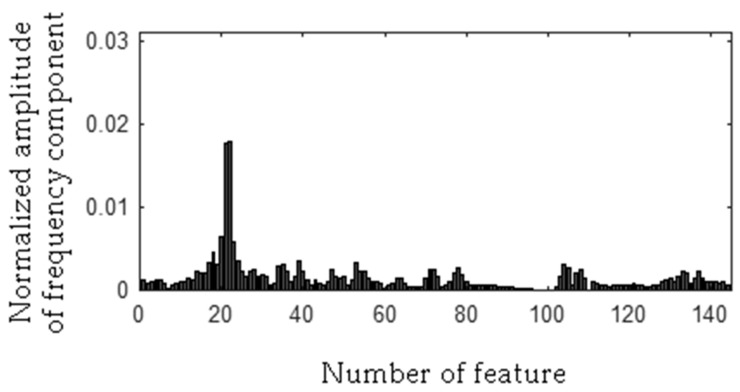
Values of features of healthy EID (145 features, seven frequency bandwidths, <271–287 Hz>, <450–490 Hz>, <550–565 Hz>, <2290–2324 Hz>, <11091–11118 Hz>, <11183–11220 Hz>, <11232–11253 Hz>).

**Figure 24:**
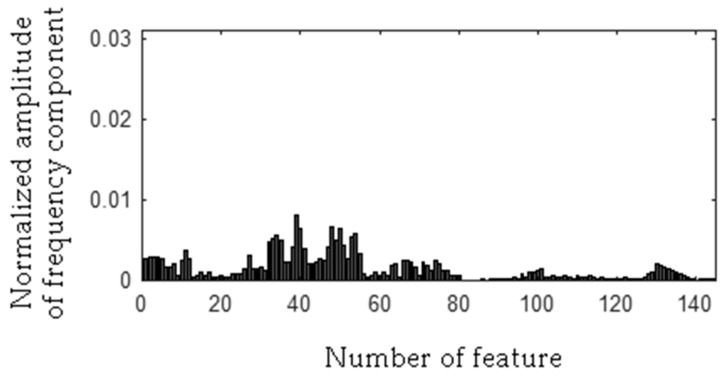
Values of features of the EID with 15 broken rotor blades (faulty fan) (145 features, seven frequency bandwidths, <271–287 Hz>, <450–490 Hz>, <550–565 Hz>, <2290–2324 Hz>, <11091–11118 Hz>, <11183–11220 Hz>, <11232–11253 Hz>).

**Figure 25:**
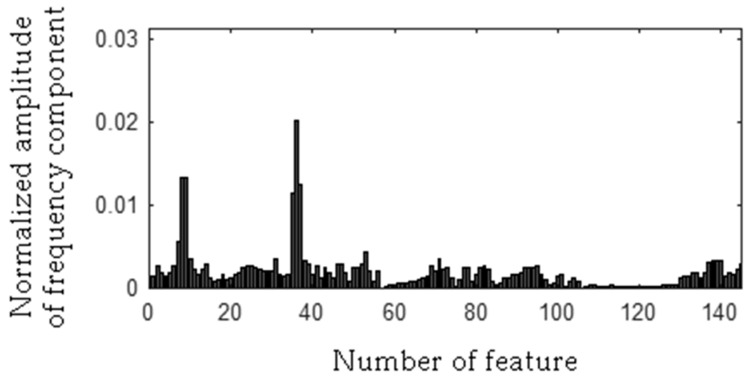
Values of features of the EID with a bent spring (145 features, seven frequency bandwidths, <271–287 Hz>, <450–490 Hz>, <550–565 Hz>, <2290–2324 Hz>, <11091–11118 Hz>, <11183–11220 Hz>, <11232–11253 Hz>).

**Figure 26:**
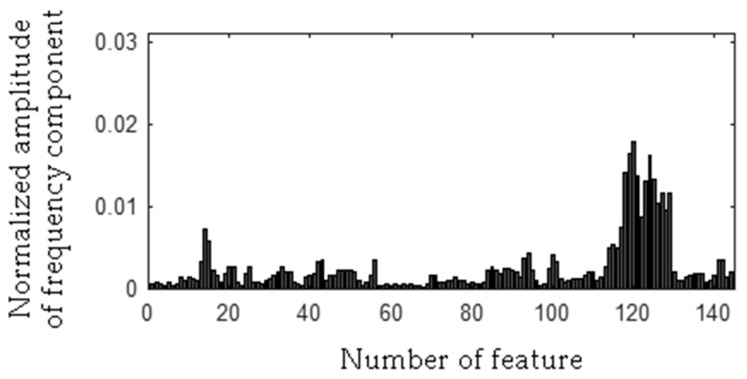
Values of features of the EID with a rear ball bearing fault (145 features, seven frequency bandwidths, <271–287 Hz>, <450–490 Hz>, <550–565 Hz>, <2290–2324 Hz>, <11091–11118 Hz>, <11183–11220 Hz>, <11232–11253 Hz>).

The frequency component 278 Hz was found, so the first frequency bandwidth is 271–285 Hz (278 − 7 Hz, 278 + 7 Hz). The MSAF-17-MULTIEXPANDED-FILTER-14 method computed frequency bandwidth 14 Hz. It can be noticed that frequency component 280 Hz is within the frequency bandwidth. Thus, the frequency bandwidth is 271–287 Hz etc. The selected frequency bandwidths/features of the EID were depicted in Figure 23, Figure 24, Figure 25 and Figure 26. The value of the parameter *ToCFCs* was equal to 0.25 for the EID.

**Figure 27:**
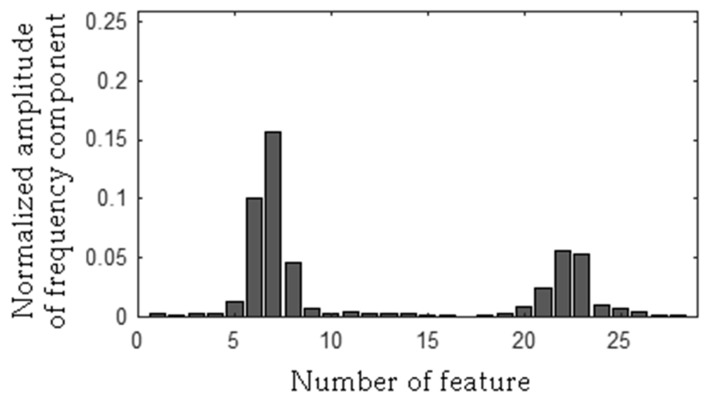
Values of features of the healthy CG-A (29 features, two frequency bandwidths, <515–537 Hz>, <1560–1575 Hz>).

**Figure 28:**
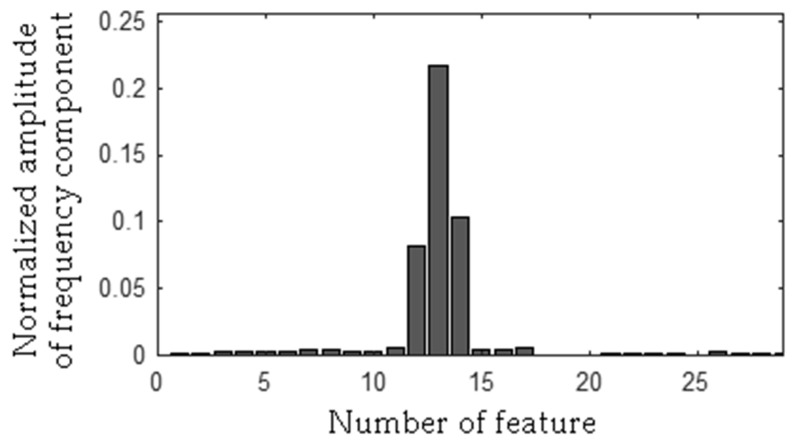
Values of features of the CG-A with a heavily damaged rear sliding bearing (29 features, two frequency bandwidths, <515–537 Hz>, <1560–1575 Hz>).

**Figure 29:**
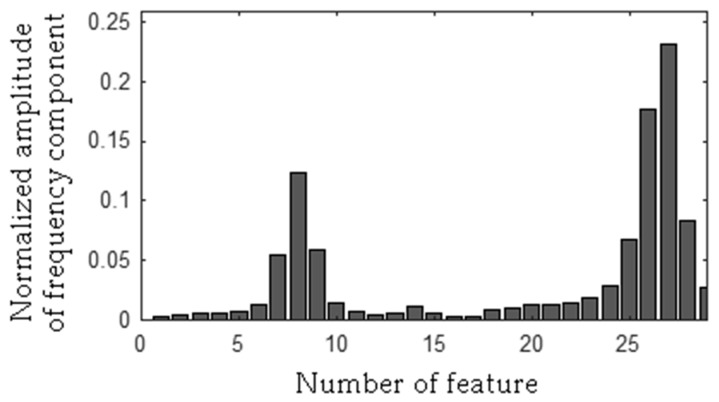
Values of features of the CG-A with a damaged shaft and heavily damaged rear sliding bearing (29 features, two frequency bandwidths, <515–537 Hz>, <1560–1575 Hz>).

The MSAF-17-MULTIEXPANDED-FILTER-14 selected two frequency bandwidths of the CG-A: <515–537 Hz>, <1560–1575 Hz>. The selected frequency bandwidths/features of the CG-A are depicted in Figure 27, Figure 28 and Figure 29. The value of the parameter *ToCFCs* was equal to 0.5 for the CG-A.

**Figure 30:**
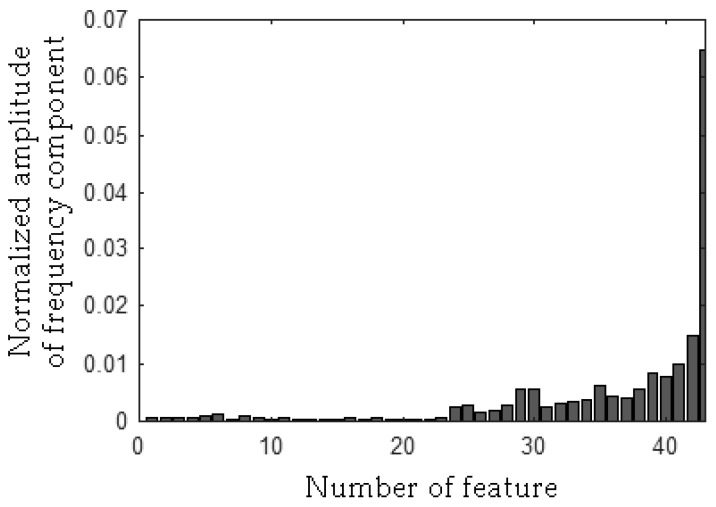
Values of features of the healthy CG-B (43 features, three frequency bandwidths, <94–109 Hz>, <194–207 Hz>, <463–488 Hz>).

**Figure 31:**
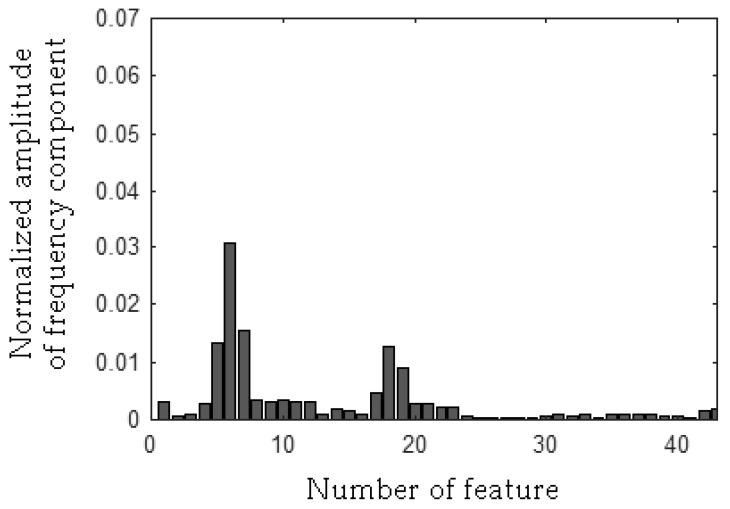
Values of features of the CG-B with a light damaged rear sliding bearing (43 features, three frequency bandwidths, <94–109 Hz>, <194–207 Hz>, <463–488 Hz>).

The MSAF-17-MULTIEXPANDED-FILTER-14 selected three frequency bandwidths of the CG-B: <94–109 Hz>, <194–207 Hz>, <463–488 Hz>. The selected frequency bandwidths/features of the CG-B are depicted in Figure 30 and Figure 31. The value of the parameter *ToCFCs* was equal to 0.5 for the CG-B.

Next computed features were classified. To classify features the NN classifier [29,30,31] was used (please see Section 2.3). There are 145 features in the feature vector. It can be noticed that distance classifiers (for example: k-means, Nearest Mean) should have also good results. Fuzzy classifiers [32] and neural network [33,34,35] can be also suitable for the acoustic-based approach. The NN classifier was selected because of its good recognition efficiency for multi-dimensional vectors. 

### 2.2. RMS

The second method of feature extraction used for the proposed acoustic based approach is the Root Mean Square (RMS). The RMS is a well-known method for feature extraction. It is defined as Equation (2):

(2) $$x_{RMS}=\sqrt{\frac{1}{n}(x_{1}^{2}+x_{2}^{2}+...+x_{n}^{2})}$$

where *x_RM_*_S_—RMS for 1-s sample (44,100 values), *n*—number of all samples, *n* = 44,100, *x*_1_, ..., *x*_*n*_—values of samples 1, ..., *n* (sampling rate 44,100 Hz).

In the presented analysis (please see Section 3) the author used 50 1-s samples for each class of the EID. Two hundred and fifty 1-s samples were used for five classes (of the EID). There were *x_RM_*_S1_, ..., *x_RM_*_S50_—RMS values of the healthy EID, *x_RM_*_S51_, ..., *x_RM_*_S100_—RMS values of the EID with 15 broken rotor blades (faulty fan), *x_RM_*_S101_, ..., *x_RM_*_S150_—RMS values of the EID with a bent spring, *x_RM_*_S151_, ..., *x_RM_*_S200_ − RMS values of the EID with a shifted brush (motor off), *x_RM_*_S201_, ..., *x_RM_*_S250_—RMS values of the EID with a rear ball bearing fault. The computed RMS values of the EID are presented in Table 1, Table 2, Table 3, Table 4 and Table 5.

The values of the RMS of acoustic signals “Healthy EID” and “EID with a rear ball bearing fault” were similar. It will be difficult to recognise these two classes. In the presented analysis (please see Section 3) the author used 50 1-s samples for each class of the CG-A. Two hundred 1-s samples were used for four classes (of the CG-A). There were *x_RM_*_S251_, ..., *x_RM_*_S300_—RMS values of the healthy CG-A, *x_RM_*_S301_, ..., *x_RM_*_S350_—RMS values of the CG-A with a heavily damaged rear sliding bearing, *x_RM_*_S351_, ..., *x_RM_*_S400_ − RMS values of the CG-A with a damaged shaft and heavily damaged rear sliding bearing, *x_RM_*_S401_, ..., *x_RM_*_S450_—RMS values of the motor off (CG-A off). The values *x_RM_*_S401_, ..., *x_RM_*_S450_ were the same as RMS values of the EID with a shifted brush (EID off). The computed RMS values of the CG-A are presented in Table 6, Table 7 and Table 8.

The values of the RMS of acoustic signals “CG-A with a heavily damaged rear sliding bearing” and “CG-A with a damaged shaft and heavily damaged rear sliding bearing” were similar. It will be difficult to recognise these two classes.

In the presented analysis (please see Section 3) the author used 50 1-s samples for each class of the CG-B. One hundred and fifty 1-s samples were used for three classes (of the CG-B). There were *x_RM_*_S451_, ..., *x_RM_*_S500_—RMS values of the healthy CG-B, *x_RM_*_S501_, ..., *x_RM_*_S550_—RMS values of the CG-B with a light damaged rear sliding bearing, *x_RM_*_S551_, ..., *x_RM_*_S600_—RMS values of the motor off (CG-B off). The values *x_RM_*_S551_, ..., *x_RM_*_S600_ were the same as RMS values of the EID with a shifted brush (EID off). The computed RMS values of the CG-B are presented in Table 9 and Table 10.

### 2.3. NN Classifier

The NN classifier is very known in the literature [29,30,31]. This type of a classifier is based on lazy learning. It does not generalize the training data. Each training feature vector has a label with a class (ID of the class). The label (ID of the class) is given to the feature vector in the training phase.

An unlabeled test feature vector is used in the classification (testing) phase. The NN classifier assigns the label, which is the closest to the training data. For this reason, distance metric is used. The author used Euclidean distance, although other distance functions could be used. Similar results were obtained using other distance functions (Manhattan distance and Minkowski distance). Euclidean distance was defined as Equation (3):

(3) $$ED\left(x-y\right)=\sqrt{\overset{n}{\underset{i=1}{\sum }}|{\left(x_{i}-y_{i}\right)}^{2}|}$$

where **x**—test feature vector, **y**—training feature vector, *ED*(**x**−**y**)—Euclidean distance, *n*—number of features (it is 1 feature for the RMS).

The NN classifier is useful for classification of feature vectors. It was found application in pattern recognition, speaker recognition, image recognition, text recognition, face recognition etc. The NN classifier is described in detail in [29,30,31].

## 3. Recognition Results of the EID, CG-A, CG-B

The analysed EID was powered from the 230 V/50 Hz mains. The author used 50G515 electric impact drills. Other devices could be used. It generated five acoustic signals denoted as: healthy EID, EID with 15 broken rotor blades (faulty fan), EID with a bent spring, EID with a shifted brush (motor off), EID with a rear ball bearing fault. Measurements were carried out in the room 3 m × 3 m. The analysed EID had rated power *P_D_* = 500 W, rotation speed *R_D_* = 3000 rpm and weight *M_D_* = 1.84 kg.

The analysed CG-A was also powered from the 230 V/50 Hz mains. The author used a ME-1498 coffee grinder. Other devices could be used. The analysed CG-A consisted of a FY5420 motor (rated power 140 W). It had rotor speed of 28,000–30,000 rpm. It generated four acoustic signals denoted as: healthy, with a slightly damaged rear sliding bearing, with a moderately damaged rear sliding bearing, motor off.

The analysed CG-B was also powered from the 230 V/50 Hz mains. The author used a SCG 1050WH coffee grinder. The analysed CG-B consisted of a HC5420 motor (rated power 150 W). It had a rotor speed of 11,300 rpm. It generated three acoustic signals denoted as: healthy, with a light damaged rear sliding bearing, motor off. 

Patterns were computed using 32 training samples of the EID, 24 training samples of the CG-A, and 24 training samples of the CG-B. Each training sample had 44,100 values. The results of recognition were computed using 250 test samples of the EID, 200 test samples of the CG-A and 150 test samples of the CG-B. Test samples had the same audio parameters (sampling rate 44,100 Hz, single channel) as training samples.

The efficiency of the proposed approach was evaluated using Equation (4). This Equation (4) defined the efficiency of recognition of the EID (*E_D_*):

(4) $$E_{D1}=\left(N_{D1}\right)/\left(N_{ALL-D1}\right)\cdot 100\%$$

where: *E*_*D*1_—the efficiency of recognition for D1 class (in the analysis it is one of five classes, for example healthy EID), *N*_*D*1_—the number of test samples classified as D1 class, *N*_*ALL-D*1_—the number of all test samples in D1 class. The values of *E_CG-A_* and *E_CG-B_* were computed similarly to *E*_*D*1_.

The total efficiency of recognition of all classes (*TE_D_*) was also introduced. It was defined as follows Equation (5):

(5) $$TE_{D}=\left(E_{D1}+E_{D2}+E_{D3}+E_{D4}+E_{D5}\right)/5$$

where *TE_D_*—the total efficiency of recognition of all classes (five states of the EID), *E_D_*_1_—the efficiency of recognition for D1 class (in the presented analysis D1 class—healthy EID), *E_D_*_2_—the efficiency of recognition for D2 class (in the presented analysis D2 class—EID with a bent spring), *E_D_*_3_—the efficiency of recognition for D3 class (in the presented analysis D3 class—EID with 15 broken rotor blades), *E_D_*_4_—the efficiency of recognition for D4 class (in the presented analysis D4 class—EID with a shifted brush), *E_D_*_5_—the efficiency of recognition for D5 class (in the presented analysis D5 class—EID with a rear ball bearing fault). The values of *TE_CG-A_* and *TE_CG-B_* were computed similarly to *TE_D_*. Four acoustic signals were used for *TE_CG-A_*. Three acoustic signals were used for *TE_CG-B_*. The computed values of *E_D_* and *TE_D_* were presented in Table 11 and Table 12. Acoustic signals of the EID were processed by the MSAF-17-MULTIEXPANDED-FILTER-14 method and the NN classifier (Table 11).

Acoustic signals of the EID were processed by the RMS and NN classifier (Table 12).

The computed values of *E_D_* and *TE_D_* of the proposed approach were following: *E_D_* = 88–100%, *TE_D_* = 96% for the MSAF-17-MULTIEXPANDED-FILTER-14 method and *E_D_* = 56–100%, *TE_D_* = 83.2% for the RMS. The computed values of *E_CG-A_* and *TE_CG-A_* were presented in Table 13 and Table 14. Acoustic signals of the CG-A were processed by the MSAF-17-MULTIEXPANDED-FILTER-14 method and the NN classifier (Table 13).

Acoustic signals of the CG-A were processed by the RMS and NN classifier (Table 14).

The computed values of *E_CG-A_* and *TE_CG-A_* of the proposed approach were following: *E_CG-A_* = 88–100%, *TE_CG-A_* = 97% for the MSAF-17-MULTIEXPANDED-FILTER-14 method and *E_CG-A_* = 92–100%, *TE_CG-A_* = 96% for the RMS. The computed values of *E_CG-B_* and *TE_CG-B_* were presented in Table 15 and Table 16. Acoustic signals of the CG-B were processed by the MSAF-17-MULTIEXPANDED-FILTER-14 method and the NN classifier (Table 15).

Acoustic signals of the CG-B were processed by the RMS and NN classifier (Table 16).

The computed values of *E_CG-B_* and *TE_CG-B_* of the proposed approach were following: *E_CG-B_*= 100%, *TE_CG-B_* = 100% for the MSAF-17-MULTIEXPANDED-FILTER-14 method and RMS.

## 4. Discussion

The acoustic-based fault-detection technique is significant for the recent research area of electrical motors. This approach is useful for inspection of motor condition. It can analyse acoustic signals in places with limited or no access. The novelty of the proposed work was to detect faults of an EID and two coffee grinders. The author focused on feature extraction of five acoustic signals of the EID, four acoustic signals of the CG-A and three acoustic signals of the CG-B. The method MSAF-17-MULTIEXPANDED-FILTER-14 was developed and described. One of the difficulties to solve was selection of training samples. It can be noticed that the recognition results depended on selected training samples. All samples is measured by one microphone. If the acoustic signal is measured by another type of microphone, then it can cause errors of recognition. The proposed acoustic-based approach should use one type of microphone for training as well as testing.

The second of the difficulties to solve was the testing (classification) of a new unknown test samples. It is difficult to recognize, for example, the acoustic signal of a car if we have training samples of an EID. To solve this problem the proposed acoustic-based approach used the NN classifier. The NN classifier found the nearest feature vector (analysed frequency bandwidths). If the acoustic signal of the car is measured, then it will be recognised as an unknown state of the EID. The training set consisted of acoustic signals of the EID and several unknown sounds of cars, ships, helicopters, animals, etc.

It can be noticed that the RMS was very good for recognition of acoustic signals of the EID with a shifted brush (motor off). This class of acoustic signal should be detected by the RMS. However, the RMS method was not good for similar sound intensity level values. The classes of acoustic signals “Healthy EID” and “EID with a rear ball bearing fault” had low values of *TE_D_*. The classes of acoustic signals “CG-A with a heavily damaged rear sliding bearing” and “CG-A with a damaged shaft and heavily damaged rear sliding bearing” had lower values of *TE_CG-A_*. The MSAF-17-MULTIEXPANDED-FILTER-14 method was good method of feature extraction for all analysed classes of acoustic signals.

## 5. Summary and Conclusions

This paper presented fault-detection techniques for an electric impact drill (EID), coffee grinder A (CG-A), and coffee grinder B (CG-B) using acoustic signals. Measurements of the acoustic signals of the EID, CG-A, and CG-B were carried out using a microphone. Five signals of the EID were analysed: healthy EID, EID with 15 broken rotor blades (faulty fan), EID with a bent spring, EID with a shifted brush (motor off), EID with a rear ball bearing fault. Four signals of the CG-A are analysed: healthy CG-A, CG-A with a heavily damaged rear sliding bearing, CG-A with a damaged shaft and heavily damaged rear sliding bearing, motor off. Three acoustic signals of the CG-B are analysed: healthy CG-B, CG-B with a light damaged rear sliding bearing, motor off.

Methods such as RMS, MSAF-17-MULTIEXPANDED-FILTER-14 were used for feature extraction. The MSAF-17-MULTIEXPANDED-FILTER-14 was also developed and described in the paper. The classification is carried out using the Nearest Neighbour (NN) classifier. An acoustic based analysis was carried out. The computed values of *E_D_* and *TE_D_* of the proposed approach were following: *E_D_* = 88–100%, *TE_D_* = 96% for the MSAF-17-MULTIEXPANDED-FILTER-14 and *E_D_* = 56–100%, *TE_D_* = 83.2% for the RMS. The computed values of *E_CG-A_* and *TE_CG-A_* of the proposed approach were following: *E_CG-A_* = 88–100%, *TE_CG-A_* = 97% for the MSAF-17-MULTIEXPANDED-FILTER-14 method and *E_CG-A_* = 92–100%, *TE_CG-A_* = 96% for the RMS. The computed values of *E_CG-B_* and *TE_CG-B_* of the proposed approach were following: *E_CG-B_* = 100%, *TE_CG-B_* = 100%.

The acoustic-based analysis was inexpensive. The experimental setup consisted of a microphone and computer. It cost about $500. Pros of this solution are instant measurement and online monitoring of the motor. Cons of this solution are the higher cost and size of the computer. The developed acoustic-based approach has many applications, for example in home and industrial appliances for fault detection. It can be used for electrical motors, engines, machinery and electric power tools [36,37,38,39,40,41,42]. It can also find applications in mining, oil, car, energy, and the steel industry. It can analyse acoustic signals in places with limited or no access. However, the proposed acoustic-based approach has one limitation. It cannot work for a machine that does not generate acoustic signals. Background noises can be also problem, if we analyse several motors in one place and at the same time.

In the future, the proposed acoustic-based approach can be further developed. Other faults of commutator motors can be added to an acoustic signal database. Measurements can be carried out using acoustic cameras and microphone arrays. Vibration-based methods can be added to the fault detection system of commutator motors. New feature extraction methods can also be developed in the future.

## Figures and Tables

**Table 1:** RMS values of the healthy EID.

Number of Samples	RMS Value	Number of Samples	RMS Value
x_RMS1_	0.237122	x_RMS5_	0.240819
x_RMS2_	0.231192	x_RMS6_	0.236356
x_RMS3_	0.234878	x_RMS7_	0.239650
x_RMS4_	0.238282	x_RMS8_	0.238406

**Table 2:** RMS values of the EID with 15 broken rotor blades (faulty fan).

Number of Samples	RMS Value	Number of Samples	RMS Value
x_RMS51_	0.322252	x_RMS55_	0.312347
x_RMS52_	0.316197	x_RMS56_	0.318529
x_RMS53_	0.317383	x_RMS57_	0.310883
x_RMS54_	0.305535	x_RMS58_	0.302719

**Table 3:** RMS values of the EID with a bent spring.

Number of Samples	RMS Value	Number of Samples	RMS Value
x_RMS101_	0.250579	x_RMS105_	0.245578
x_RMS102_	0.244888	x_RMS106_	0.243813
x_RMS103_	0.244461	x_RMS107_	0.246395
x_RMS104_	0.249611	x_RMS108_	0.246297

**Table 4:** RMS values of the EID with a shifted brush.

Number of Samples	RMS Value	Number of Samples	RMS Value
x_RMS151_	0.006427	x_RMS155_	0.006478
x_RMS152_	0.006338	x_RMS156_	0.007226
x_RMS153_	0.008981	x_RMS157_	0.007020
x_RMS154_	0.009021	x_RMS158_	0.006644

**Table 5:** RMS values of the EID with a rear ball bearing fault.

Number of Samples	RMS Value	Number of Samples	RMS Value
x_RMS201_	0.235278	x_RMS205_	0.234696
x_RMS202_	0.236730	x_RMS206_	0.236078
x_RMS203_	0.233518	x_RMS207_	0.237600
x_RMS204_	0.234478	x_RMS208_	0.237778

**Table 6:** RMS values of the healthy CG-A.

Number of Samples	RMS Value	Number of Samples	RMS Value
x_RMS251_	0.203343	x_RMS255_	0.209252
x_RMS252_	0.203521	x_RMS256_	0.215012
x_RMS253_	0.201109	x_RMS257_	0.209241
x_RMS254_	0.205511	x_RMS258_	0.205984

**Table 7:** RMS values of the CG-A with a heavily damaged rear sliding bearing.

Number of Samples	RMS Value	Number of Samples	RMS Value
x_RMS301_	0.234359	x_RMS305_	0.234927
x_RMS302_	0.234860	x_RMS306_	0.233882
x_RMS303_	0.231783	x_RMS307_	0.235229
x_RMS304_	0.237120	x_RMS308_	0.229835

**Table 8:** RMS values of the CG-A with a damaged shaft and heavily damaged rear sliding bearing.

Number of Samples	RMS Value	Number of Samples	RMS Value
x_RMS351_	0.239449	x_RMS355_	0.248779
x_RMS352_	0.246317	x_RMS356_	0.250027
x_RMS353_	0.246894	x_RMS357_	0.250791
x_RMS354_	0.247325	x_RMS358_	0.250203

**Table 9:** RMS values of the healthy CG-B.

Number of Samples	RMS Value	Number of Samples	RMS Value
x_RMS451_	0.248146	x_RMS455_	0.248331
x_RMS452_	0.254812	x_RMS456_	0.259062
x_RMS453_	0.248951	x_RMS457_	0.263240
x_RMS454_	0.240446	x_RMS458_	0.264600

**Table 10:** RMS values of the CG-B with a lightly damaged rear sliding bearing.

Number of Samples	RMS Value	Number of Samples	RMS Value
x_RMS501_	0.131587	x_RMS505_	0.103367
x_RMS502_	0.121155	x_RMS506_	0.095910
x_RMS503_	0.103567	x_RMS507_	0.108105
x_RMS504_	0.094650	x_RMS508_	0.105756

**Table 11:** Computed values of *E_D_* and *TE_D_* of the EID using the MSAF-17-MULTIEXPANDED-FILTER-14 method and the NN classifier.

**Type of Acoustic Signal**	**E_D_ (%)**
Healthy EID	100
EID with a bent spring	92
EID with (15 broken rotor blades) faulty fan	100
EID with shifted brush (motor off)	100
EID with rear ball bearing fault	88
	**TE_D_ (%)**
Total efficiency of recognition of the EID	96

**Table 12:** Computed values of *E_D_* and *TE_D_* of the EID using the RMS and the NN classifier.

**Type of Acoustic Signal**	**E_D_ (%)**
Healthy EID	56
EID with a bent spring	100
EID with (15 broken rotor blades) faulty fan	100
EID with shifted brush (motor off)	100
EID with rear ball bearing fault	60
	**TE_D_ (%)**
Total efficiency of recognition of the EID	83.2

**Table 13:** Computed values of *E_CG-A_* and *TE_CG-A_* of the CG-A using the MSAF-17-MULTIEXPANDED-FILTER-14 method and the NN classifier.

**Type of Acoustic Signal**	**E_CG-A_ (%)**
Healthy CG-A	100
CG-A with a heavily damaged rear sliding bearing	100
CG-A with a damaged shaft and heavily damaged rear sliding bearing	88
Motor off	100
	**TE_CG-A_ (%)**
Total efficiency of recognition of the CG-A	97

**Table 14:** Computed values of *E_CG-A_* and *TE_CG-A_* of the CG-A using the RMS and the NN classifier.

**Type of Acoustic Signal**	**E_CG-A_ (%)**
Healthy CG-A	100
CG-A with a heavily damaged rear sliding bearing	92
CG-A with a damaged shaft and heavily damaged rear sliding bearing	92
Motor off	100
	**TE_CG-A_ (%)**
Total efficiency of recognition of the CG-A	96

**Table 15:** Computed values of *E_CG-B_* and *TE_CG-B_* of the CG-B using the MSAF-17-MULTIEXPANDED-FILTER-14 method and the NN classifier.

**Type of Acoustic Signal**	**E_CG-B_ (%)**
Healthy CG-B	100
CG-B with a light damaged rear sliding bearing	100
Motor off	100
	**TE_CG-B_ (%)**
Total efficiency of recognition of the CG-B	100

**Table 16:** Computed values of *E_CG-B_* and *TE_CG-B_* of the CG-B using the RMS and the NN classifier.

**Type of Acoustic Signal**	**E_CG-B_ (%)**
Healthy CG-B	100
CG-B with a light damaged rear sliding bearing	100
Motor off	100
	**TE_CG-B_ (%)**
Total efficiency of recognition of the CG-B	100
